# Congenital hereditary endothelial dystrophy - mutation analysis of *SLC4A11* and genotype-phenotype correlation in a North Indian patient cohort

**Published:** 2010-12-31

**Authors:** Preeti Paliwal, Arundhati Sharma, Radhika Tandon, Namrata Sharma, Jeewan S. Titiyal, Seema Sen, Tapas C. Nag, Rasik B. Vajpayee

**Affiliations:** 1 Laboratory of Cyto-Molecular Genetics, Department of Anatomy, All India Institute of Medical Sciences, New Delhi, India; 2 Dr. Rajendra Prasad Centre for Ophthalmic Sciences, All India Institute of Medical Sciences, New Delhi, India; 3 Electron microscope facility, Department of Anatomy, All India Institute of Medical Sciences, New Delhi, India; 4 Centre for Eye Research Australia, University of Melbourne, Australia

## Abstract

**Purpose:**

To identify the solute carrier family 4 (sodium borate cotransporter) member 11 (*SLC4A11*) mutation spectrum and to perform genotype-phenotype correlations in autosomal recessive Congenital Hereditary Endothelial Dystrophy (CHED2) in North Indian patients.

**Methods:**

Twenty-five patients from twenty families clinically diagnosed with autosomal recessive CHED2 were recruited for the study. Clinical parameters such as age at onset, presentation, and pre- and post-operative visual acuities were recorded. Corneal buttons of patients undergoing keratoplasty were analyzed for histopathologic and ultrastructural confirmation. All the affected individuals and 50 unrelated population matched normal controls were screened for underlying sequence changes. Genomic DNA was isolated from peripheral blood samples and all the exons and the 5′-upstream region of the *SLC4A11* gene were screened for mutations by direct DNA sequencing.

**Results:**

A high degree of consanguinity (9 out of 20 families) was noted. Corneal haze was reported to be present since birth or shortly thereafter in all affected patients. Histology and electron microscopy studies revealed increased thickness of Descemet’s membrane, especially of the non-banded zone. Molecular studies revealed one novel homozygous in-frame deletion mutation in two affected siblings from one family and three other previously reported homozygous mutations in 12 patients from 9 families. Mutations were not identified in 11 patients from 11 families. High interfamilial and intrafamilial phenotypic variability was seen among the cohort of patients.

**Conclusions:**

This is the first report on the mutation spectrum and genotype-phenotype correlation in CHED2 patients from North India. The present study detected one novel and three reported changes, adding to the repertoire of mutations in *SLC4A11,* and recorded a high degree of genetic heterogeneity in CHED2.

## Introduction

Congenital hereditary endothelial dystrophy (CHED) is defined as a rare inheritable disorder of the corneal endothelium characterized by bilateral, symmetric, non-inflammatory corneal clouding (edema) seen at the time of birth or shortly thereafter [1]. The degeneration of the corneal endothelium leads to corneal edema, especially of the stroma, and gives the cornea an appearance resembling ground glass. Two subtypes of CHED are known – autosomal dominant (CHED1; OMIM 121700) and autosomal recessive (CHED2; OMIM 217700) – based on differences in the mode of inheritance [2]. Clinically, both forms have similar presentation, the latter being more severe. Different loci have been mapped to 20p11.2-q11.2 [3] and 20p13, for CHED1 and CHED2, respectively [4].

The Solute Carrier family 4 (sodium borate cotransporter) member 11 (*SLC4A11*; OMIM 610206) has been identified as the candidate gene for CHED2 [5]. This gene has 19 exons that encode the bicarbonate transporter–related protein 1 (BTR1) of 891 amino acids and 14 transmembrane domains with intracellular amino and carboxyl terminals. Several functions have been defined for BTR1; the important ones are its role in maintenance of boron homeostasis, cell growth and proliferation, and activation of the mitogen-activated protein kinase pathway [6]. Vithana et al. [5] hypothesized that a deregulation of the mitogen-activated protein kinase pathway and a failure of the mutant protein to reach the cell surface to perform its function leads to the symptoms of CHED2 [5]. Mutations in *SLC4A11* are described in Harboyan syndrome, also known as Corneal Dystrophy and Perceptive Deafness (CDPD*;* OMIM 217400), which is characterized as CHED2 with hearing loss [7].

Several studies have been performed to analyze the role of this gene in causation of CHED2, which involves mainly patients from South India [8-10]. There are no published data available from the Northern part of India. Therefore, the present study was undertaken to assess the role of *SLC4A11* in the pathogenesis of CHED2 in North Indian patients.

## Methods

The study protocol adhered to the tenets of Declaration of Helsinki and the Institutional Ethics Committee provided ethical approval for the study. In total, 25 patients from 20 families diagnosed with CHED2 were included in the study. Corneal dystrophy was diagnosed on clinical examination. Detailed family histories were collected and pedigree charts were constructed for all of the patients; the presence of consanguinity was noted based on marriage histories.

### Clinical studies

The clinical examination included routine slit lamp biomicroscopy, confocal microscopy, specular microscopy, ultrasonic pachymetry, orbscan, and ultrasonography (USG) for posterior segment evaluation. Patients who were bilaterally affected and without any other systemic involvement formed the study group. The diagnosis of CHED was made on the basis of the following characteristics: presence of mosaic corneal haze with corneal edema, increased central corneal thickness (>0.7 mm in all cases), normal horizontal corneal diameter (10–11 mm), and no evidence of congenital glaucoma (e.g., no buphthalmos, Haab’s striae, or optic disc cupping).

The control population consisted of 50 healthy, unrelated, population-matched individuals who had no history of any ocular disease in their family.

### Histological studies

Histopathology and electron microscopic (EM) studies were performed on 7 corneal buttons obtained from 7 patients after penetrating keratoplasty surgery. Each corneal button was divided in half; one half was used for histopathology (fixed in 10% buffered formalin) and the other half for electron microscopic (EM) studies.

For histopathological studies, the formalin-fixed corneal tissue samples from 7 patients were embedded in paraffin blocks and cut into 4 μM thick sections. Sections were analyzed by light microscopy after staining with hematoxylin and eosin (H&E), periodic acid Schiff, and Congo red dyes.

### Ultrastructural analysis

The corneal tissues were collected from five patients at the time of penetrating keratoplasty and immediately fixed in Karnovski fixative. Two corneal tissue samples could not be processed for transmission electron microscopy as the patients had undergone keratoplasty 8 years previously.

After the dehydration and clearing processes, the tissue samples were embedded in araldite blocks. Sections were cut with an ultramicrotome (UC-6; Leica; Lab India, New Delhi, India) using a glass knife and the electron micrographs were taken with an electron microscope (MORGAGNI 268D; FEI, Eindhoven, The Netherlands).

### Genetic analysis

A 5 ml sample of peripheral blood was collected by venipuncture in EDTA from all patients and controls after taking informed consent from all of the participants. Genomic DNA was extracted by the salting out method [11].

The DNA was amplified in a thermocycler (ABI 9700; Applied Biosystems [ABI], Foster City, CA) by PCR using the primers as described previously (Table 1) [5]. The amplification reaction mixture consisted of 10 ng DNA, 1.5 mM MgCl_2_, 0.25 mM of each dNTP, 10pM of each primer, and 0.7 units of Taq polymerase (Roche, ABI, Foster City, CA), and Q-sol (Qiagen GmBH, Hilden, Germany) in a total volume of 25 μl. The amplified PCR products were subjected to gel purification using QIAmp gel extraction kits (Qiagen GmBH) and the purified PCR products were screened for sequence changes by bidirectional sequencing. The amplified products were sequenced directly with BigDye Terminator Mix version 3.1 (ABI) according to the manufacturer’s instructions and were then analyzed on an ABI-3100 Genetic Analyzer (ABI). Nucleotide sequences for the coding regions were compared with the nucleotide sequence of the published *SLC4A11* human cDNA (NM_032034). The families that did not show *SLC4A11* coding region changes were screened for mutations in the putative promoter region using primers described earlier [8].

**Table 1:** Table showing primer sequences and PCR conditions used for amplification of *SLC4A11* and *CHST6* genes.

***SLC4A11* gene**			
** **	**Primer Sequence 5′to 3′**		** **
**Exons**	**Forward**	**Reverse**	**Annealing temperature (°C)**
1	CCTAGCAGATGGGCTAAGCA	GAGCAAAGCCACAGGACTCT	60
2 and 3	CGAGAGTGGGACAGTCCAG	CTCCCTGTTGAGTGCTCCT	62
4 and 5	TCCAGGAGCAGCTCAACAG	CAGCCCTCTTCTCCCAAGTT	57
6	CCAACCAACTTGGGAGAAGA	CCTTCAGAGGCCAGGACAT	52
7 and 8	AAAACCTGCTGCCAGTTCAT	CCTAGGAATGGGGGATGG	57
9 and 10	ACTGATGGTACGTGGCCTCT	CGTCCATGCGTAGAAGGAGT	58
11 and 12	TCTACATCCAGGGTGCAGTG	CGTCCATGCGTAGAAGGAGT	56
13 and 14	GAGCCCTTTCTCCCTGAGAT	GGTTGTAGCGGAACTTGCTC	61
15 and 16	CGGGAAATCGAGAGTGAGTT	CGTCTCCTTCACGTTCACAA	54
17 and 18	CTGGCCACATGGGACATAG	CTAGGCAGGACCCCTCCTC	53.5
19	CAGGAGGGGCTCCAGTCTA	CTGTCCCTTGCATTCCACTT	55
Putative Promoter region 1	GCCTTACTCACCCAATCTATGC	CCCTGTCTCCTCCTTTCGAC	61
Putative Promoter region 2	GGAGGAGGAGAAGGACTTGC	GCACACTCGCGCACTCAC	55
***CHST6* gene**			
** **	**Primer Sequence 5′to 3′**		** **
**Coding region**	**Forward**	**Reverse**	**Annealing temperature**
1	GCCCCTAACCGCTGCGCTCTC-	GGCTTGCACACGGCCTCGCT	57
2	GACGTGTTTGATGCCTATCTGCCTTG-	CGGCGCGCACCAGGTCCA	55
3	CTCCCGGGAGCAGACAGCCAA	CTCCCGGGCCTAGCGCCT	57

One family was screened for underlying changes in the carbohydrate (N-acetylglucosamine 6-O) sulfotransferase 6 (*CHST6*; OMIM 605294) gene as the mother was suspected to have macular corneal dystrophy based on her phenotypic presentation. PCR amplification was done using the primers as described previously (Table 1) [12] in a reaction mixture consisting of 100 ng DNA, 1.5 mM MgCl_2_, 0.25 mM of each dNTP, 10pM of each primer, and 1.0 units of Taq polymerase (Roche, ABI), and Q-sol (Qiagen GmBH) in a total volume of 25 μl.

### Polyphen 2 and SIFT analysis

The PolyPhen 2 and SIFT (Sorting Intolerant From Tolerant) tools were used for analysis of novel mutations to characterize the pathogenic nature of the identified changes. The SIFT tool generates multiple sequence alignments of a gene over different species and assesses the degree of conservation of the substituted positions over the course of evolution. It gives a value as a score, where a score <0.05 is considered to represent a potentially damaging mutation.

## Results

Several clinical parameters were assessed to confirm autosomal recessive CHED2 in the patients forming the study group.

### Clinical examinations

Corneal pachymetry revealed increased corneal thickness in all the cases. The age of the patients ranged from 4 to 34 years (Table 2). The intraocular pressure was within normal range (below 21 mmHg) for all patients. Nystagmus was present in 19 patients and 38 eyes. A high degree of consanguinity (9 out of 20 families) was noted among the CHED2 patients. Corneal haze was reported to have been present since birth or shortly thereafter in all the cases. Clinical examinations (slit lamp biomicroscopy) of parents (thirty parents from seventeen families) revealed unaffected clear corneas. None of the patients in the present study group had associated sensorineural hearing loss.

**Table 2:** Details of families with autosomal recessive congenital hereditary endothelial dystrophy and *SLC4A11*mutations.

** **	** **	** **	** **	** **	** **	**BCVA at last F/Up**		** **	** **	** **	** **	** **	** **	** **
**S. no.**	**Family no.**	**Age/ sex**	**Nystagmus**	**CCT RE/LE**	**Age at PK**	**RE**	**LE**	**Histopathological findings**	**Complications**	**Consanguinity**	**Exon affected**	**c.DNA position of the change**	**Amino acid position**	**Indian state of origin**
1	Q1	16/F	P	PE/PE	ND	CF	CF	-	-	P	16 (Splice site)	c.2240+1G>A	Inactivation of splice site	U.P.
2		18/M	A	980/1010	18	6/24	CF	ED+DMT	-					
3	Q2	18/F	P	PE/PE	10	6/24	CF	ED+ DMT	Graft rejection	A	18	c.2470G>A	Val824Met	U.P.
4		22/M	P	PE/PE	12	CF	CF	ED+ DMT	-					
5	Q3	22/F	P	PE/516	20	CF	6/18	ED+ DMT	Graft rejection	A	18	c.2470G>A	Val824Met	Bihar
6		17/F	P	1058/PE	17	6/36	CF	ED+ DMT	Graft rejection					
7	Q4	7/M	P	841/921	ND	6/60	6/60	-	-	A	-	NMD	-	Bihar
8	Q5	17/M	A	PE/PE	ND	CF	6/24	-	-	P	-	NMD	-	U.P.
9	Q6	28/F	P	770/PE	26	4/60	CF	ED+ DMT	-	A	18	(**NOVEL**) c2518-c2520 delCTG	Leu840del	Haryana
10		26/M	P	PE /853	ND	2/60	CF	-	-					
11	Q7	24/M	P	1011/PE	ND	CF	6/24	-	-	A	-	NMD	-	Haryana
12	Q8	11/M	P	PE/PE	ND	CF	1/60	-	-	P	-	NMD	-	U.P.
13	Q9	14/M	A	PE /994	ND	6/60	2/60	-	-	P	-	NMD	-	Orissa
14	Q10	34/M	P	PE/PE	ND	1/60	6/60	-	-	A	-	NMD	-	U.P.
16	Q12	4/M	P	PE/PE	ND	Seeing+Fixing	Seeing+Fixing	-	-	P	9	c.1156T>C	Cys386Arg	Haryana
17	Q13	22/M	A	838/PE	ND	CF	CF	-	-	A	-	NMD	-	U.P.
18	Q14	9/M	P	PE/1023	ND	6/24	6/60	-	-	P	-	NMD	-	J&K
19	Q15	20/F	P	PE/PE	ND	CF	6/36	-	-	P	9	c.1156T>C	Cys386Arg	U.P.
20		16/F	P	920/PE	ND	CF	1/60	-	-					
21	Q16	7/F	P	PE/1008	ND	3/60	3/60	-	-	A	16	c.2470G>A	Val824Met	Punjab
22	Q17	8/M	P	PE/987	ND	1/60	1/60	-	-	A	-	NMD	-	Delhi
23	Q18	21/M	A	PE/PE	18	6/24	CF	ED+ DMT	Graft rejection	A	-	NMD	-	U.P.
24	Q19	4/M	P	PE/PE	ND	Seeing+Fixing	Seeing+Fixing	-	-	A	-	NMD	-	Bihar
25	Q20	12/F	P	PE/PE	ND	2/60	4/60	-	-	P	18	c.2470G>A	Val824Met	U.P.

PK-Penetrating keratoplasty, P- Present, A- Absent, F- Female, M- male, ND-Not Done, ED- endothelial degeneration, DMT- Descemet Membrane Thickening, NMD - No mutation Detected, UP- Uttar Pradesh, J & K- Jammu & Kashmir, IOP-Intraocular pressure, CCT- Central corneal thickness, BCVA-Best corrected visual acuity after keratoplasty, RE-Right eye, LE-Left Eye, F/up period- follow up period, PE-Error on pachymetry.

### Light microscopy analysis

Histopathological examination for the patients undergoing keratoplasty confirmed the diagnosis of CHED2 in all of the patients. Light microscopy revealed widening of the stroma and marked thickening of Descemet’s membrane. Few atrophic endothelial cells could be identified in two cases, whereas the rest of the corneal tissues had completely atrophied endothelium (Table 2).

### Ultrastructural analysis

Electron microscopy (EM) revealed degeneration of keratocytes. Fragmentation and disorganization of collagen fibers was seen, leading to separation of collagen lamellae. An overall increase in stromal thickness was observed, with the presence of numerous vacuoles or water clefts in the stroma. The most conspicuous observation was an increase in the total thickness of the Descemet’s membrane, which was due to an increase in the thickness of the non-banded zone (5–8 times thicker than the normal); the banded zone had normal thickness and morphology. The non-banded zone had numerous collagen bundles, but no endothelial cells were observed, indicating almost complete absence and attenuation of the endothelium. These findings were similar for all of the CHED2 patients, in whom the ultrastructural imaging was done, confirming the clinical diagnosis.

### Mutation analysis

Direct DNA sequence analysis showed 14 CHED2 patients from 9 families had underlying *SLC4A11* mutations that co-segregated with the disease phenotype and which were absent in 50 population matched controls.

**Figure 1:**
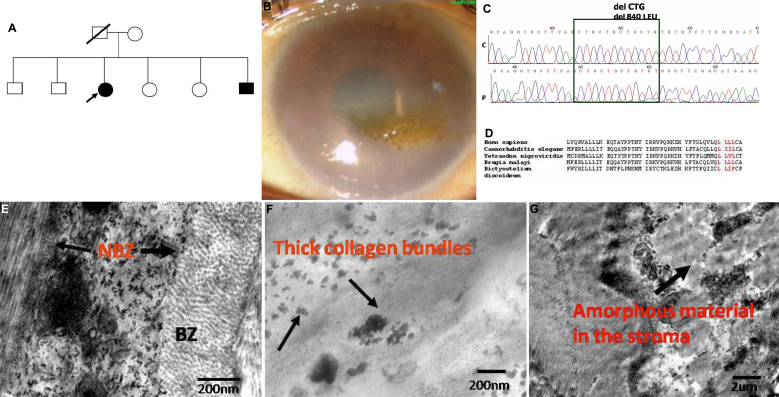
Genotype-phenotype features of the novel *SLC4A11* deletion mutation. **A**: Pedigree of the family showing a novel deletion mutation of one of the four leucine residues c2518-c2520 del CTG in the exon 18 of Solute Carrier Family 4 (sodium borate co-transporter) member 11 (*SLC4A11)*. Filled boxes represent affected individuals. Open boxes represent unaffected individuals. Arrow indicates the proband. **B**: Slit lamp photomicrographs of the affected individual harboring the novel mutation. The representative clinical photograph shows the presence of marked stromal haze and spheroidal degeneration in the right eye of the proband. **C**: Partial nucleotide sequence of *SLC4A11*. The chromatogram of the patient (P) is shown in comparison to control (C). The block marks the four CTG repeats in the control and only three in the patient. The homozygous deletion of CTG residue in the patient can be noted. **D**: Multiple sequence alignment of *SLC4A11* gene from different species. The amino acid leucine (L) at positions 840–843 is conserved over a range of species in the course of evolution, which are highlighted in red. **E**: Transmission electron micrographs of the affected patient harboring the novel mutation. **E**: Transmission electron micrograph showing Descemet’s membrane of the CHED2 patient. Descemet’s membrane is thickened, with a normal anterior banded zone and a thickened posterior banded layer (Scale bar 2 μm). **F**: This panel represents a magnified view of part of the posterior banded layer showing presence of thick collagen bundles indicated by the arrows. **G**: The disorganized corneal stroma can be noted along with the presence of amorphous material. (Scale bar 2 μm).

A novel in-frame homozygous deletion mutation of one of the four leucine residues c2518-c2520 del CTG in exon 18 was seen in two affected siblings of a family (Figure 1A) who also had associated spheroidal degeneration (Figure 1B). The presence of mutation was confirmed on bidirectional sequencing in both the siblings (Figure 1C). Conservation of the amino acid residues involved in the novel deletion mutations is shown in Figure 1D. Ultrastructural studies of the proband carrying the novel mutation revealed the presence of large vacuoles in the stromal keratocytes and a marked increase in the thickness of the Descemet’s membrane (Figure 1E-G).

**Figure 2:**
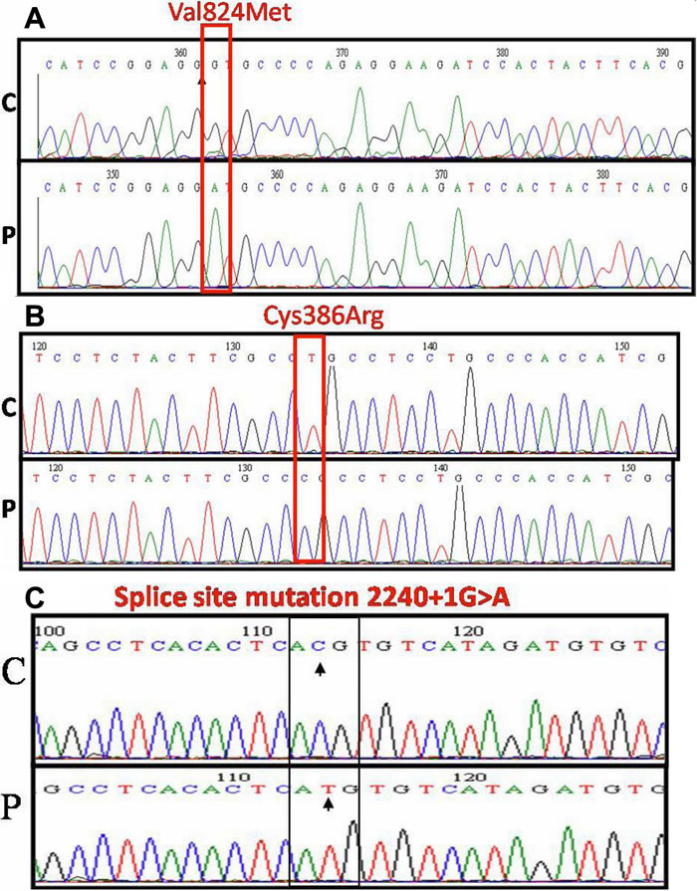
Mutations in *SLC4A11* causing CHED2. **A**: Partial nucleotide sequence *SLC4A11*. The chromatograms of the patients (P) are shown in comparison to controls (C). The homozygous G >A substitution is marked by the block. The block denotes the nucleotide with a missense mutation resulting in amino acid substitution of valine at amino acid position 824 with methionine. **B**: The homozygous T>C substitution is marked by the block. The block denotes the nucleotide with a missense mutation resulting in amino acid substitution of cysteine at amino acid position 386 with arginine. **C**: The homozygous G>A substitution is marked by the block. The block denotes the nucleotide with a missense mutation resulting in a splice site mutation c.2240+1G>A.

Other mutations documented in the study included a homozygous missense mutation c.2470G>A in exon 18 in 6 affected patients from 4 families (Figure 2A). A homozygous missense mutation, c.1156T>C, was seen in three affected individuals from 2 families that led to substitution of a cysteine residue at amino acid position 386 with arginine (C386R; Figure 2B). Both of these mutations have been reported in patients from South India. A splice site mutation c.2240+1G>A was seen in a family that showed a variable phenotype (Figure 2C). Apart from these, two homozygous changes in introns 2 and 11 were identified in two patients from two families, respectively, but no coding region changes, either heterozygous or homozygous, were seen in them. No other coding region variations were identified in 11 patients from 11 families. In addition, screening of the upstream putative promoter region also failed to reveal any change in these 11 individuals.

### Details of the family (Q1) with a variable phenotypic presentation and a splice site mutation c.2240+1G>A

**Figure 3:**
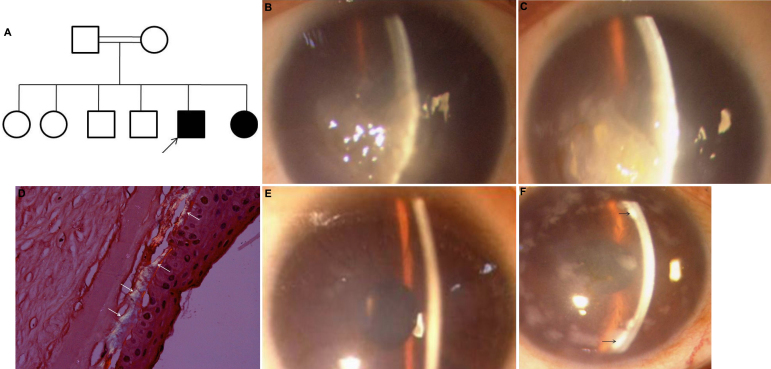
Family Q1 showing variable phenotype. **A**: Pedigree of the family showing the splice site mutation c.2240+1G>A and variable phenotypic presentation of the affected members. Filled boxes represent affected individuals. Open boxes represent unaffected individuals. Arrow indicates the proband. A double line indicates presence of consanguinity in the family. **B**, **C**: Representative slit lamp photomicrographs of the proband with a homozygous splice site mutation c.2240+1G>A. The representative clinical photographs of right (**B**) and left eye (**C**) of the proband shows the presence of the typical ground glass appearance of the cornea seen in autosomal recessive CHED. **D**: shows the presence of apple green birefringence on staining with Congo-red and viewing under polarized filter, marked by arrows. **E**: The slit lamp photomicrograph of the right eye of the affected sibling had marked stromal haze. **F**: The clinical photomicrograph of the mother shows the endothelial deposits (marked by arrows) with stromal haze. A few epithelial deposits are also seen.

The proband, a 20 year old male (Figure 3A), presented with bilateral ground glass cornea with bullae. Corneal cloudiness and nystagmus were reported to have been present right from birth (Figure 3B,C). Family history revealed parental consanguinity and one more affected sibling. Visual acuity of the proband was counting fingers from a 1 m distance in the right eye and counting fingers close to the face in the left eye. Corneal pachymetry revealed increased corneal thickness that was beyond the measurement limits of the specular microscope. Molecular analysis identified a homozygous splice site mutation c.2240+1G>A.

Histopathological analysis identified a markedly thickened Descemet’s membrane and an atrophied endothelium, confirming the diagnosis of CHED2. Additionally, the patient’s cornea also had amyloid deposition and spheroidal degeneration. The presence of amyloid was confirmed based on the presence of apple green birefringence when viewed under a polarizing filter (Figure 3D).

The affected sibling, i.e., a younger sister of the proband, was also examined. She was 15 years old, with cloudy corneas since birth, but no nystagmus. Bilateral diffuse limbus-to-limbus stromal haze with stromal thickening and guttae changes was seen (Figure 3E). Vision in both eyes was limited to counting fingers from 1 m. Corneal pachymetry revealed an increased corneal thickness (right eye- 980, left eye-1010). Molecular analysis identified a homozygous splice site mutation c.2240+1G>A. None of the unaffected siblings showed the mutation in either homozygous or heterozygous state.

The 60-year-old mother was also clinically examined and she reported to have had a history of bilateral lime injury during childhood. Defective vision, photophobia, and watering had been present for the past 15 years (Figure 3F). Vision in the right eye was recorded as counting fingers from 1 m distance and the left eye had vision restricted to counting fingers close to the face. Corneal pachymetry revealed a normal corneal thickness (right eye-524µm and left eye-527 µm) but bilateral endothelial opacities and stromal haze were noted. Additionally, a few epithelial opacities were seen. The phenotype of the mother resembled macular corneal dystrophy with epithelial and endothelial involvement. Mutation analysis identified the heterozygous splice site mutation c.2240+1G>A. No other changes were noted in the coding region, overlapping splice sites, and the putative promoter region of *SLC4A11*.

In view of the different clinical presentation seen in the mother, who resembled macular corneal dystrophy, the affected family members were screened for mutations in the *CHST6* gene, which are known to cause macular corneal dystrophy. No coding region changes were seen in *CHST6,* either in the mother or in any of her affected offspring.

## Discussion

The *SLC4A11* gene codes for BTR1 that functions as a sodium borate co-transporter (NaBC1) and plays a role in activation of the mitogen activated protein kinase pathway [6]. Mutations in the *SLC4A11* gene lead to nonsense mediated mRNA decay or formation of a truncated protein that is unable to reach the surface and perform its function. It is the subsequent deregulation of the MAPKinase pathway that is thought to cause CHED2.

In the present study we identified a novel c2518-c2520 delCTG mutation and assessed its pathogenic nature using SIFT and PolyPhen 2 tools. The deleted leucine residue lies in the transmembrane domain 11 and in silico analysis showed that the leucine residues at all the positions in the transmembrane domain, ranging from 840 to 843, are conserved over a wide range of species. Deletion of any one of them can disrupt the appropriate assembly or localization of the protein within the membrane. This may consequently have lead to CHED2 in the two siblings, although the exact mechanism remains unknown.

The splice site mutation c.2240+1G>A, identified in family Q1 with CHED2 affects the first nucleotide of the splice donor site and may probably cause mis-splicing of the pre-mRNA transcript. This would lead to either, exon skipping or intron retention, which would consequently result in an altered protein structure. However, it is difficult to determine the exact nature of the mis-splicing and the fate of the mutant transcripts, due to non-expression of *SLC4A11* in lymphocytes [13]. Gene expression studies to determine the nature and variable expression of *SLC4A11* would require corneal tissue of heterozygous individuals, which was not possible in the present study.

The most interesting feature of family Q1 was the intrafamilial variability with presence of variable phenotypes in the affected siblings who were homozygous for the splice site mutation and a completely different phenotypic presentation in the mother who was a heterozygous carrier. Other family members who were heterozygous carriers of the same mutation were unaffected and had clear corneas. The mutation c.2240+1G>A has been reported as the single heterozygous change leading to CHED2 in a British white (Caucasian) from the UK (UK), while the same change was not observed in 30 South Indian and 50 European controls of the study [13]. Similarly, in the present study, this change was not seen in the 50 controls also screened for *SLC4A11* mutations. The reason for the presence of corneal opacities in some heterozygous carriers and not in others is not clear. The most plausible explanation could be that it is an autosomal dominant change with variable penetrance and expressivity. This change could also be described as a rare polymorphism segregating with the disease phenotype in the family. However presence of the same change in heterozygous condition in a CHED2 patient of British origin is suggestive of it being a pathogenic mutation.

Other previously reported mutations that were seen included c.2470G>A in 6 affected patients from 4 families and c.1156T>C in 3 affected individuals from 2 families. Both the mutations showed interfamilial and/or intrafamilial variability and no genotype-phenotype correlation was seen. Similar studies from South India have also reported absence of evident correlations between clinical and histopathologic findings, and *SLC4A11* mutations [9,14]. These reports implicate that phenotype presentation depends not only on the underlying mutation but also involves role of other genes, developmental and/or environmental factors.

In the present study, mutations were seen in 14 affected individuals from 9 families in a cohort of 25 patients. Several reports have been published describing the role of *SLC4A11* mutations in the causation of dystrophies. In reports by Sultana et al. [9], Jiao et al. [10], and Hemadevi et al. [8], mutations in *SLC4A11* were identified in 35 of 42 (83 percent), 12 of 16 (75 percent), and 11 of 20 (55 percent) CHED2 families screened. These studies involved Indian patients, but most of them were from South India. We found mutations in 14 of 25 (56 percent) patients diagnosed with CHED2 in North India. A decrease in the number of patients with underlying mutations in *SLC4A11* in North India can be due to the difference in origin of these two populations and a relatively low rate of consanguinity in the Northern part as compared to South. The disease, being autosomal recessive, is associated with a high rate of consanguinity and thus is seen to occur at a higher frequency in the South Indian population.

Cases of CDPD are reported to have *SLC4A11* mutations that result in an alteration of gene expression in the stria vascularis causing impairment in the hearing efficiency of these individuals [7]. However none of the patients in the present study group had associated sensorineural hearing loss.

Heterozygous changes in *SLC4A11* have been reported in cases of late onset Fuchs endothelial corneal dystrophy (FECD; OMIM 136800) in which haploinsufficiency and accumulation of aberrantly folded protein is reported to result in FECD pathology [15]. The onset of symptoms for CHED2 and FECD are different, yet they share a common feature of an abnormal posterior non-banded zone of the Descemet’s membrane which points to endothelial dysfunction starting in the late prenatal period [16]. In view of this, the parents of the affected patients, who were identified as heterozygous carriers of the *SLC4A11* changes were clinically examined by slit lamp biomicroscopy. No signs and symptoms of FECD or associated guttae changes were seen in any of the parents examined, whose age range varied from 45 to 72 years.

Posterior Polymorphous Corneal Dystrophy (PPCD; OMIM 122000) is another dystrophy which results from primary endothelial dysfunction inherited as a dominant trait. Incomplete penetrance and de novo mutations have been observed in PPCD cases with identified zinc finger E-box binding homeobox 1(*ZEB1*; OMIM 189909) mutations which can lead to consideration of recessive inheritance. A few PPCD cases have been reported to present with corneal haze at birth [16] which might result in an overlap in phenotypic presentation of CHED and PPCD. Considering this, CHED2 cases with no *SLC4A11* coding region changes should be screened for mutations in *ZEB1*.

The studies on endothelial dystrophies i.e., CHED, FECD, and PPCD suggest that all these forms can actually be allelic variants of the same disease continuum and that genetic interaction between genes that cause corneal dystrophies can modulate the expressivity of the phenotype [17]. Moreover, they all are considered to represent defects of terminal differentiation of the neural crest cells [17] and share common features of disease manifestation like endothelial metaplasia, and secretion of an abnormal Descemet’s membrane [17]. Considering this variability in expression, all cases of endothelial dystrophies should be screened for the presence of *SLC4A11* and/or *ZEB1* changes for confirmation and categorization.

In conclusion, to the best of our knowledge, this is the first report of mutation screening for the *SLC4A11* gene in CHED2 cases from North India. We identified 1 novel and 3 previously reported mutations in 14 individuals from 9 families and documented inter- and intrafamilial variability, apart from the presence of genetic heterogeneity in our cohort of patients.
